# Placental Chemokine Receptor D6 Is Functionally Impaired in Pre-Eclampsia

**DOI:** 10.1371/journal.pone.0164747

**Published:** 2016-10-25

**Authors:** Chiara Tersigni, Fiorella Di Nicuolo, Giuseppe Maulucci, Alessandro Rolfo, Domenica Giuffrida, Manuela Veglia, Marco De Spirito, Giovanni Scambia, Tullia Todros, Nicoletta Di Simone

**Affiliations:** 1 Department of Obstetrics and Gynaecology, Fondazione Policlinico Agostino Gemelli, Università Cattolica Del Sacro Cuore, Rome, Italy; 2 Institute of Physics, Università Cattolica Del Sacro Cuore, Rome, Italy; 3 Department of Surgical Sciences, Sant’Anna Hospital, Università degli Studi di Torino, Turin, Italy; Chinese Academy of Sciences, CHINA

## Abstract

**Background:**

Pre-eclampsia (PE) is a major cause of maternal and perinatal morbidity and mortality worldwide. It is defined by new onset of hypertension and proteinuria after the 20^th^ week of gestation and characterized by systemic exaggerated inflammatory response. D6 is a chemokines scavenger receptor that binds with high affinity CC chemokines, internalizes and targets the ligands for degradation. It is expressed in trophoblast-derived tissues and prevents excessive placenta leukocyte infiltration.The aim of this study was to investigate the expression and function of D6 in human placentae from pre-eclamptic and healthy pregnant women.

**Methods and Results:**

Plasma levels of D6-binding CC chemokines (CCL-2, CCL-3, CCL-4, CCL-7, CCL-11) and pro-inflammatory cytokines (IL-6, TNF-α, CRP) were analyzed in 37 healthy pregnant women and 38 patients with PE by multiplex bead assay. Higher circulating levels of CCL7, CCL11, IL-6, (p<0.0001) and CRP (p<0.05) were observed in PE women compared to controls. Levels of circulating CCL4 were decreased in PE (p<0.001), while no significant differences of CCL2, CCL3 or TNF-α levels were detected. Immunofluorescent staining of placental sections showed higher expression of D6 receptor in the PE syncytiotrophoblast. Confocal and Western blot (WB) analyses revealed a prevalent distribution of D6 in trophoblast cells membranes in PE. Increased activation of D6 intracellular pathway was observed by Western blot analyses of p-LIMK and p-cofilin in trophoblast cell lysates. D6 functional assays showed reduced scavenging of CCL2 in PE cells compared to controls. Since actin filaments spatial assembling is essential for D6 intracellular trafficking and scavenging activity, we investigated by confocal microscopy trophoblast cytoskeleton organization and we observed a dramatic disarrangement in PE compared to controls.

**Conclusions:**

our results suggest membrane distribution of D6 receptor on trophoblast cell membranes in PE, together with reduced functionality, probably due to cytoskeleton impairment.

## Introduction

Pre-eclampsia (PE) is a pregnancy-specific hypertensive disorder defined as new onset hypertension and proteinuria at or after 20 weeks’ gestation [1]. Complicating 2–8% of all pregnancies, PE is a major cause of maternal morbidity and mortality and of adverse perinatal outcomes [2]. The underlying causes remain unclear but it is recognized to be a placenta-driven disorder associated with poor placental perfusion causing hypoxia-reperfusion injury and oxidative syncytiotrophoblast stress. Release into the maternal circulation of placental pro-inflammatory and anti-angiogenic factors ensues, leading to endothelial dysfunction, exaggerated maternal inflammatory response and hypercoagulability [3–5].

The systemic inflammatory response occurring in overt PE involves leukocytes, the clotting and complement systems and the endothelium. Communication between these various components of the inflammatory network is facilitated by a large variety of secreted proteins such as cytokines. Among these, chemokines are essential for leukocyte chemoattraction [6,7].

Chemokines promote leukocytes recruitment to sites of infection and inflammation by activating conventional G protein-coupled receptors [8,9]. They are also recognized by a set of atypical chemokine receptors (ACRs) that cannot induce directional cell migration but are required for the generation of chemokine gradients in tissues. ACRs are considered "silent receptors" because no G protein-dependent signaling activity is observed after their engagement by cognate ligands [10,11].

D6 decoy receptor is one of the ACRs. It binds most inflammatory, but not homeostatic, CC chemokines, internalizes constitutively, and targets the ligand for degradation [12,13].

In resting conditions, D6 is predominantly located in intracellular/perinuclear compartments and only 5% is detectable on the cell surface [14,15]. After chemokines binding, D6 is constitutively internalized and then targeted to early endosomes [12,16]. Once D6 has been internalized, ligands dissociate from the receptor and are targeted to degradation in lysosomal compartments, while the receptor is free to recycle back to the cell surface [15–17] with mechanisms that are strictly dependent on cytoskeleton dynamics [18].

Indeed, the engagement of D6 receptor by its ligands activates a β-arrestin1-dependent G protein-independent signaling pathway, the Rac1-p21-activated kinase 1 (PAK1)-LIM kinase 1 (LIMK1) cascade [18]. This cascade results in the phosphorylation and inactivation of a major actin-depolymerizing factor, cofilin, that enable actin network rearrangements that are critically required for the increased abundance of D6 protein on the cell surface and for its chemokine-scavenging activity [18].

Differently from other chemokine receptors, D6 expression has been reported mainly in non-hematopoietic cells and includes endothelial cells lining afferent lymphatic in skin, gut, and lung [19]. D6 expression has been also detected in the human placenta [20], particularly concentrated toward the apical surface of chorionic villous trophoblast, the side directly contacting maternal blood [21].

Recently, a role for D6 decoy receptor activity in the maintenance of controlled inflammatory placental environment at maternal-fetal interface has been proposed by Martinez de la Torre and co-authors, who showed that trophoblast cells express D6 and use this molecule to scavenge inflammatory CC chemokines [22]. Intriguingly, they also provided evidences that D6 is required to prevent excessive placenta leukocyte infiltration and inflammation- and autoantibody-induced fetal loss in animal models, thus protecting the fetus from miscarriage [22]. Consistently, Madigan et al. have demonstrated that, in normal pregnancy, despite robust expression of pro-inflammatory chemokines by gestational tissues, D6-binding chemokines are less abundant in maternal plasma compared to non-pregnant women. Indeed, maternal blood continuously flows towards D6-expressing chorionic villi, suggesting a crucial role for D6 decoy receptor in blood chemokines scavenging and regulation of local and systemic inflammation [21].

Conversely, a trend of increase in circulating D6-binding chemokines was observed in the third trimester in 34 women later developing PE compared to gestational age-matched controls [21].

Since PE is a placental-induced inflammatory disorder characterized by higher circulating blood levels of pro-inflammatory cytokines, like CCL2, IL-6, IL-8, TNF-α, RANTES (or CCL5) and MIF (macrophage migration inhibitory factor) [21, 23–27], the aim of this study was to investigate a possible abnormal expression or function of placental D6 receptor, and thus, of a pivotal regulatory system of tissue inflammatory response, in human placentae obtained from women with PE compared to women with physiological pregnancy.

With this purpose, we collected blood and placentae from 37 pre-eclamptic and 38 healthy pregnant women at delivery and we quantified circulating pro-inflammatory cytokines (IL-6, TNF-α, CRP) and chemokines, specifically scavenged by D6 receptor (CCL-2, CCL-3, CCL-4, CCL-7, CCL-11). Placental lysates and placental sections were investigated for D6 expression by Western blot analysis and immunofluorescent staining, respectively. Primary trophoblast cells obtained from PE women and controls were analyzed for D6 cellular distribution by confocal microscopy and Western blot analysis. Furthermore, we investigated the functional activity of D6 in trophoblast cells by both binding and scavenging assays of CCL-2 (D6-binding chemokine).Finally, we analyzed trophoblast actin fibers organization, whose integrity is crucial for D6 internalization and scavenging.

In the complex, our data indicates that D6 is concentrated on trophoblast cell membranes in PE, in line with higher circulating levels of D6-ligand chemokines, but its scavenging activity is affected by trophoblast cytoskeleton disarrangement.

## Materials and Methods

### Patients and Samples

Maternal blood samples (5 ml) from healthy pregnant women (n = 38) or PE (n = 37) were collected by vein puncture just before delivery and spun at 3000g at 4°C for 20 minutes. The supernatant were then aliquoted and immediately stored at -80°C until use.

Placentae were collected from 10 women with normal pregnancies undergoing caesarean section for breech presentation, and 10 women with PE immediately after delivery. None of the women recruited in this study were in labor at the time of placental sampling and all had singleton pregnancies with no known fetal abnormalities. Women with diabetes, autoimmune diseases or infections or sepsis were excluded from the study.

Placental biopsies were taken for being either mechanically disrupted by sonication and stored in Hepes Lysis Buffer at -20°C or embedded in parafolmadehyde for 12 hours and then in ethanol at R/T. Remnant placental tissues were used for primary trophoblast cultures as detailed below.

These studies were approved by the Ethics Committee of the Catholic University of Sacred Heart, Rome, Italy and have been conducted according to the delclaration of Helsinki. Informed written consent was obtained from all participants.

### Multiplex Bead Assay

Plasma levels of pro-inflammatory cytokines (IL-6, TNF-α, CRP) and D6-binding chemokines (CCL-2, CCL-3, CCL-4, CCL-7, CCL-11) were analyzed using a Multiplex Bead Array System, Procarta^®^ Immunoassay Kit (Diametra, Milan, Italy), according to manufacturer’s instructions. The analyses of levels of cytokines were made using the Luminex 100 instrument (Luminex Corp., Austin, TX, USA) and STarStation software (V1.1, Applied Cytometry Systems, Sheffield, UK).

### Immunofluorescent Staining

Placental sections were deparaffinised by washing for 1 minute each in Histo clear 1 and Histo clear 2 solutions before being rehydrated in a series of washes in 100% (x2), 95%, 90%, 80%, 70% and 50% alcohol solutions. Slides were dipped in sodium citrate (VWR, UK) buffer (10mM, pH 6.0) for 10 minutes in a microwave oven, cooled at R/T for 30 minutes and then washed in PBS before being incubated in a blocking solution comprising of PBS containing 10% (v/v) FCS for 1 hour at R/T. Experimental sections were then incubated O/N at 4°C in PBS containing 1% FCS and 20μg/ml of D6 primary rat monoclonal antibody (R&D Systems). After 3 washings in 0.01% PBS-T of 10 minutes each, placental sections were stained with secondary FITC-conjugated goat anti-rat antibody (Life Technologies, UK) at 1:400 dilutions for 1 hour at R/T. Sections were then washed three times for 10 minutes each with 0.01% PBS-Tand mounted under microscopic glass coverslips in Prolong^®^ Gold Antifade Reagent and evaluated by an inverted-phase fluorescent microscope (2003; Axiover 35, Zeiss). Images were acquired with a digital camera (Nikon Coolpix 4500).

### RNA Isolation and Real Time PCR

Total RNA was isolated from control and PE placental tissues using TRIzol reagent (Life Technologies, Invitrogen, Italy) according to manufacturer’s instructions. Genomic DNA contamination was removed by DNAse I digestion before RT-PCR. cDNA was generated from 5 μg of total RNA using a random hexamers approach and RevertAid H Minus First Strand cDNA Synthesis kit (Fermentas, Italy).

Gene expressions levels of D6 were determined by Real Time PCR using specific TaqMan primers and probe (CCBP2 hs00174299 m1, Life Technologies, Italy). mRNA levels were normalized using endogenous 18s as internal reference (Life Technologies, Italy). Relative expression and fold change were calculated according to Livak and Schmittgen [28].

### Primary Trophoblast Cells Culture

Based on the characteristic syncytiotrophoblast distribution of D6 in placental sections, to investigate D6 expression in primary trophoblast cells from PE and controls (CTR), placentae were collected from 10 PE and 10 CTR patients and cytotrophoblast cells were isolated as detailed elsewhere [29]. Cells`viability, assessed by trypan blue dye exclusion, was 90%. The purity of the cell preparation was evaluated by immunohistochemical staining for markers of a) macrophages (3%, determined using a polyclonal anti-a1-chymotrypsin antibody; Dako, Santa Barbara, CA,USA); b) fibroblasts (2%, determined using a polyclonal anti-vimentin antibody; Labsystems, Helsinki, Finland); and c) syncytiotrophoblast (1% determined using a monoclonal antibody against low molecular weight cytokeratins; Labsystems, Chicago, IL,USA). The enriched (95%) trophoblast cells were cultured in Dulbecco’s modified Eagle’s medium (DMEM, Sigma-Aldrich, St. Louis, MI,USA) with 10% fetal bovine serum (FBS, Sigma) at 37°C in 5% CO_2_/95% air for 48 hours.

### Confocal Microscopy Analysis of D6 Expression in Trophoblast Cells

To quantify and localize D6, trophoblast cells were seeded on glass bottom dishes (Ibidi, Germany, 5 x 10^4^ cell/dish). After 24 hours cells were rinsed twice in PBS, fixed with 4% PFA for 10 minutes at room temperature (R/T) and, then, incubated for 1 hour at R/T with primary anti-D6 antibody (10 μg/ml of rat monoclonal anti-human D6, R&D Systems, USA) and then with secondary goat anti-rat Alexa Fluor^®^ 488 conjugated antibody (Life Technologies, UK) at 1:400 dilutions for 1 hour at R/T.

All stainings were visualized by an inverted confocal microscope (SP2 Leica Microsystems, Wetzlar, Germany) using a 40/1.25 NA oil objective and processed using LCS software version 2.61 (Leica Microsystems). Internal photon multiplier tubes collected images in 8-bit, unsigned images at a 400-Hz scan speed. PMT was kept always at the same operating voltage during the experiment. FITC was excited with an Ar/Kr laser line (excitation wavelength: 488 nm, emission range: 500–560 nm). Analysis of acquired images (fluorescence intensity evaluation on cell membranes and on the interior part of the cell) was performed with Image-J (NIH). Fluorescence values for plasma membranes were determined within multiple regions of interest (ROI) for each sample. Free hand ROIs were drawn on cell membranes in transmission images and transposed on fluorescence images for measurements as previously described [30]. Traced ROIs were characterized by a length of several microns, and by a pixel resolution width. For each sample n = 50 cells were analyzed.

### Western Blot Analysis

Placental lysates and cell lysates were obtained by sonication. Plasma membranes (post-nuclear particulate fraction without cytosolic components) from trophoblast cells were isolated as described elsewhere [31]. Briefly, cell pellet was resuspended in 2,5 ml of ice-cold buffer (250 mM sucrose, 20 mM HEPES, pH 7.4, 2 mM EGTA, 3 mM NaN3) containing freshly added protease inhibitors (200 μM pheylmethylsulphonylfluoride and 1 μM leupeptin; Sigma) and homogenized in a 5ml glass Dounce homogenizer. The homogenate was centrifuged at 700 g for 5 min to remove nuclei and unbroken cells and the resultant supernatant was centrifuged at 236,000 x g for 60 min to pellet cell membranes. The membranes were resuspended in homogenizing buffer and frozen at -80°C until use. Protein concentration was measured by BCA.

For Western blotting, eighty μg of placenta, trophoblast cell or membrane lysates from PE or CTR were separated by 10% SDS–PAGE electrophoresis under reducing conditions. After gel electrophoresis and transfer of proteins to a nitrocellulose membrane, nitrocellulose sheets were blocked at R/T for 1 h in 5% non-fat dry milk. Placental lysates were incubated overnight at +4°C with rat primary anti human D6 antibody (dilution 1:1000; Santa Cruz Biotechnology, Santa Cruz, CA, USA) and mouse anti-actin antibody (dilution 1:500; Thermo Fisher Scientific, Waltham, MA, USA) as loading control. Trophoblast cell lysates were incubated overnight at +4°C with anti human D6 antibody and mouse anti human Glyceraldehyde-3-phosphate dehydrogenase (GAPDH) antibody (dilution 1:1000; Abcam, Cambridge, UK), a cytoplasmic protein. Membrane lysates were incubated overnight at +4°C with anti D6 antibody, anti GAPDH antibody and anti human E-Cadherin antibody (dilution 1:1000; Cell Signaling Technology Inc., Danvers, MA, USA).), a plasma membrane marker. For intracellular D6 pathway investigation, trophoblast cell lysates only were incubated with 5 μg/ml of specific primary mouse anti p-LIMK [thr508] or anti p-Cofilin [ser3] antibody (Cell Signaling Technology Inc., Danvers, MA, USA).

After incubation with primary antibodies, membranes were washed with PBST and incubated in specific horseradish peroxidase-conjugated secondary antibody diluted 1:2000 in 5% non-fat dried milk in PBST. Following incubation with secondary antibody, the immuno-complexes were visualized with ECL-Plus detection System (Amersham Biosciences Corp. USA) according to manufacturer's instruction. Bands were analyzed using the Gel Doc 200 System (Bio-Rad Laboratories, Milan, Italy) and quantified by Quantity One quantitation Software (Bio-Rad).

### CCL2-D6 Binding Assay

Trophoblast cells obtained from PE and control placentae were seeded on glass bottom dishes (Ibidi, Germany, 5 x 10^4^ cell/dish) and incubated, according to manufacturer’s instructions,with biotinylated human CCL2 (R&D systems) for 60 min at 2–8°C. Avid-FITC reagent for additional 30 min at 2–8°C in the dark was added. Cells were then rinsed three times in 1X PBS and visualized by an inverted confocal microscope (SP2; Leica Microsystems, Germany) with a 63x oil immersion objective (NA 1.4). Z-planes were acquired every 300 nm from the bottom to the top of cells. Spot counting was performed on every plane by using image-J (NIH), and the total number of spots was calculated for each cell as described elsewhere [32].

### D6 Scavenging Assay

Trophoblast cells obtained from PE and control placentae were plated the day before the experiment in 96-well plates (5 × 10^4^ cells/well) and then incubated with 10 ng/mL of recombinant CCL2 (D6 ligand, R&D Systems) for 12 or 24 hrs. The supernatant was collected and the chemokine concentration was evaluated at different times of incubation (0-12-24 hrs) by sandwich ELISA (R&D Systems) according to manufacturer’s instructions.

### F-Actin Immunofluorescent Staining

To investigate trophoblast cells cytoskeleton organization, cells from PE or control placentae, primary cell cultures were seeded on glass bottom dishes (Ibidi, Germany, 5 x 10^4^ cell/dish) in DMEM with 10% FBS. After cells were rinsed twice in PBS, fixed with 4% PFA for 10 min at R/T, and permeabilized with 0.1% Triton-X for 5 min. Next, cells were incubated with Phalloidin Fluorescein Isothiocyanate Labeled according to manufacturer’s instructions for 30 min at R/T (Sigma-Aldrich, USA). F-actin fibers were visualized by an inverted confocal microscope (SP2; Leica Microsystems) with a 63x oil immersion objective (NA 1.4).

### Statistical Analysis

Means and Standard Deviation (SD) or Medians and Minimum-Maximum ranges were used to describe quantitative variables whereas absolute and relative frequencies were employed for categorical ones. Data from *in vitro* experiments were analysed using one-way analysis of variance (ANOVA) followed by a *post-hoc* test (Bonferroni test). The results are shown as the mean ± standard error (SE). For all analyses, p<0.05 was considered significant. Cytokines plasma levels and clinical characteristics of PE and CTR women were compared by Chi square test or Mann Whitney test according to the type of variables. P value <0.05 was considered statistically significant.

## Results

The clinical characteristics of PE patients and healthy pregnant women enrolled in this study are reported in Tables 1 and 2. Pre-eclamptic women showed, as expected, higher body mass index (BMI) (p<0.001), lower gestational age at delivery (p<0.001) and lower neonatal birth weight (p<0.001) compared to controls.

**Table 1:** Characteristics of patients enrolled in the study.

Characteristics	Pre-eclampsia (n = 37)	Control (n = 38)	P
Age (years)	34 (24–44)**	33 (21–44)**	0.54
Ethnic group			
• Caucasian	35 (94.6%)	32 (84.2%)	0.19
• Afro-Caribbean	2 (5.4%)	6 (15.8%)	
Nulliparous	24 (64.9%)	17 (44.7%)	0.10
BMI at booking (Kg/m^2^)	25.8 (7.2)*	22.2 (4.6)*	<0.001
Preeclampsia onset			
• Early	14 (41.2%)	N/A	
• Late	20 (58.8%)	N/A	
Uterine artery Doppler velocimetry			
• Normal	12 (32.4%)		
• Abnormal	24 (64.9%)		
• Not performed	1 (2.7%)	38 (100%)	
Gestational age at delivery (weeks)	34 (23–41)**	40 (37–41)**	<0.001
Birth weight (g)	1650 (970)*	3450 (360)*	<0.001
• SGA	18 (48.6%)	0 (0.0%)	<0.001
• AGA	19 (51.4%)	38 (100.0%)	

*Mean (Standard Deviation).

**Median (Minimum-Maximum); N/A: not applicable. SGA: small for gestational age. AGA: appropriate for gestational age.

**Table 2:** Clinical details of the patients who donated placentas for *in vitro* studies.

Patients	Gestation(weeks+days)	Parity	Age(years)	Maximum BP(mmHg)	Proteinuria(mg/dL)	Birth Weight(g)	Percentile(°)
Normal pregnant partecipants							
N1	39+1	0+0	28	125/80	N/D	3180	53
N2	39+3	2+0	31	120/80	N/D	3760	84
N3	39+4	0+0	35	115/70	N/D	3480	68
N4	39+1	1+0	34	110/80	N/D	2940	33
N5	39+2	1+0	32	120/80	N/D	3500	78
N6	39+0	0+0	32	125/80	N/D	3250	58
N7	39+3	0+0	28	110/70	N/D	3100	37
N8	39+5	2+0	26	120/80	N/D	3660	83
N9	39+3	0+0	29	110/80	N/D	2800	22
N10	39+5	0+0	34	110/80	N/D	3360	59
Pre-eclamptic partecipants							
PE1	37+0	0+0	32	140/95	1.8	2240	10
PE2	39+3	0+0	29	160/100	0.5	2940	23
PE3	31+5	0+0	34	180/110	1.5	1300	10
PE4	33+2	1+0	35	160/100	1.0	1700	17
PE5	37+4	1+0	37	200/130	1.9	1990	8
PE6	22+1	0+0	27	170/110	1.0	350	-
PE7	33+2	0+0	27	155/100	3.5	1490	10
PE8	39+3	0+0	32	145/105	0.4	3560	75
PE9	30+0	0+3	38	130/100	0.5	910	9
PE10	36+4	0+0	24	145/95	0.7	3000	59

N: normal pregnant women. PE: pre-eclamptic women. BP: blood pressure. N/D: not detected. N/P: not performed.

### Pre-Eclamptic Patients Show Higher Plasma Levels of D6-Binding Chemokines

Higher circulating levels of chemokines CCL7 (p<0.001) and CCL11 (p<0.001) as well as cytokines IL-6 (p<0.001) and CRP (p<0.05) were observed in pre-eclamptic women compared to healthy pregnant women (p<0.001). Levels of circulating CCL4 were found to be lower in PE women compared to controls (p<0.001) while no significant differences were observed in terms of CCL2, CCL3 or TNF-α(p = 0.7, p = 0.4 and p = 0.05, respectively) blood levels between PE and healthy pregnant women (Table 3).

**Table 3:** Circulating levels of D6-binding chemokines and pro-inflammatory cytokines in women with pre-eclampsia and in healthy pregnant women.

Chemokines	Pre-eclampsia (n = 37)	Control (n = 38)	P
CCL2 (pg/ml)	15.6 (3.2–65.4)	15.9 (1.3–39.8)	0.7
CCL3 (pg/ml)	1.9 (0.3–5.7)	1.9 (0.3–8.2)	0.4
CCL4 (pg/ml)	230.6 (17.8–2086.7)	292.9 (43.1–827.8)	<0.001
CCL7 (pg/ml)	22.9 (4.6–180.4)	4.6 (4.6–70.4)	<0.001
CCL11 (pg/ml)	33.4 (0.6–84.7)	20.7 (3.3–56.7)	<0.001
IL-6 (pg/ml)	1.5 (0.4–21.7)	0.7 (0.2–9.7)	<0.001
TNF-α (pg/ml)	0.3 (0.3–7.5)	0.2 (0.3–8.5)	0.05
CRP (mg/ml)	3.6 (63.9–0.3)	1.2 (36.6–0.3)	<0.05

Luminex assays revealed significantly increased levels of CCL7, CCL11, IL-6 and CRP, and decreased levels of CCL4 in serum samples of PE women *versus* controls (CTR). Results are expressed as median (minimum-maximum range) of four experiments. P value <0.05 was considered statistically significant.

### D6 Decoy Receptor Shows a Syncytiotrophoblast Distribution in placentae from Pre-Eclamptic Women

**Fig 1:**
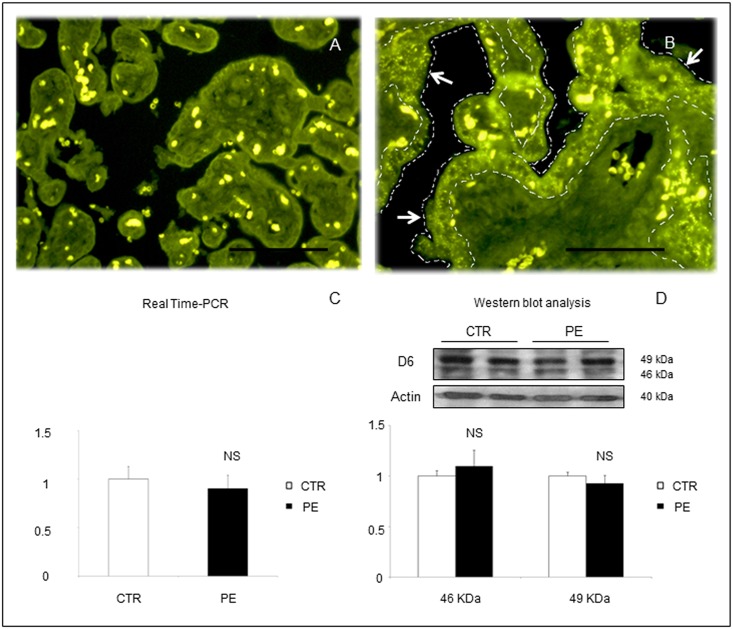
Representative images of immunofluorescent staining for D6 in placental sections from (A) a normal pregnant woman at term or (B) a woman with PE at 28 weeks of gestation. Characteristic positive expression for D6 receptor was detected in the syncytiotrophoblast monolayer in PE (B). Scale bar 100 μm. No significant differences were found between PE and control placental lysates in terms of overall D6 tissue expression by RT-PCR (C) or Western blot analysis (D). Results are expressed as mean ± SE of six experiments. NS: not significant.

Immunofluorescent staining for D6 decoy receptors of placental sections obtained revealed a more intense staining in placentas obtained from women with PE compared to CTR with a characteristic distribution in syncytiotrophoblast cells monolayer surrounding the chorionic villi (Fig 1A and 1B).

### Real Time PCR and WB Analysis of D6 in Placental Lysates

Quantitative analysis of D6 decoy receptor transcription or expression of whole placental lysates did not show any significant difference between CTR and PE (Fig 1C and 1D).

### WB Analysis of D6 in Trophoblast Cell Cultures

Quantitative analysis of D6 decoy receptor expression in lysates of primary trophoblast cells cultured for 48 h did not show any significant difference between CTR and PE (S1 Fig).

### D6 Decoy Receptor Displays a Membrane Distribution in Trophoblast Cells in PE

**Fig 2:**
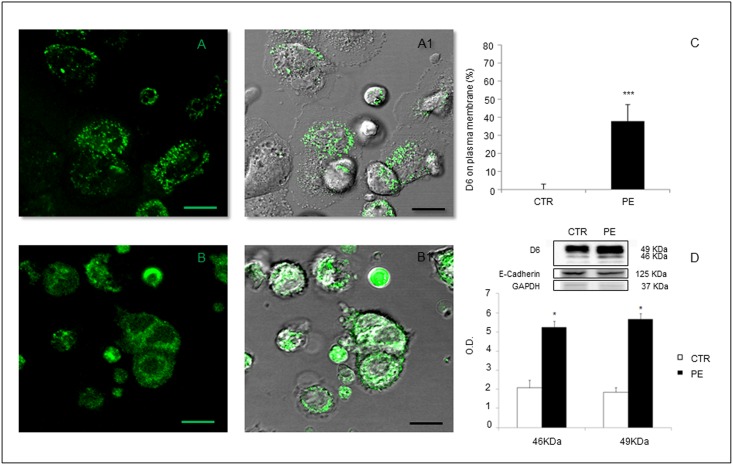
Representative image of D6 expression in primary trophoblast cell cultures from normal pregnant women (A and A1) or a women with PE (B and B1) assessed by confocal microscopy. Cells from PE patients showed a more intense expression of D6 (green) on cell membranes together with a more diffuse cytoplasm distribution compared to control cells, which displayed atypical speckled cytoplasmic D6 positivity. A and B: D6 fluorescent staining. A1 and B1: D6 overlay between fluorescent staining and electronic transmission. Scale bar 20 μm. Quantification of D6 expression on trophoblast cell membranes was performed by (C) confocal analysis of fluorescence on membranes, as percentage of the whole cells fluorescence, and (D) Western blot analysis of D6 on cell membrane lysates. Expression of both 46 and 49 KDa isoforms of D6 was increased in trophoblast membranes in PE. Results are expressed as mean ± SE of six experiments.*p<0.05; ***p<0.001.

As shown in Fig 2, in trophoblast cells from CTR, D6 receptor was mainly localized in intracellular compartments (Fig 2, panels **A** and **A.1**), while in PE D6 showed a striking higher distribution in trophoblast membranes (Fig 2, panels **B** and **B.1** and S2 Fig). Fig 2C and 2D. Quantitative analysis of D6 expression by confocal microscopy and WB analysis confirmed higher concentration of D6 on trophoblast cell membranes in PE compared to controls.

### D6 Intracellular Pathway Is Over-Activated in Trophoblast Cells from PE Placentae

**Fig 3:**
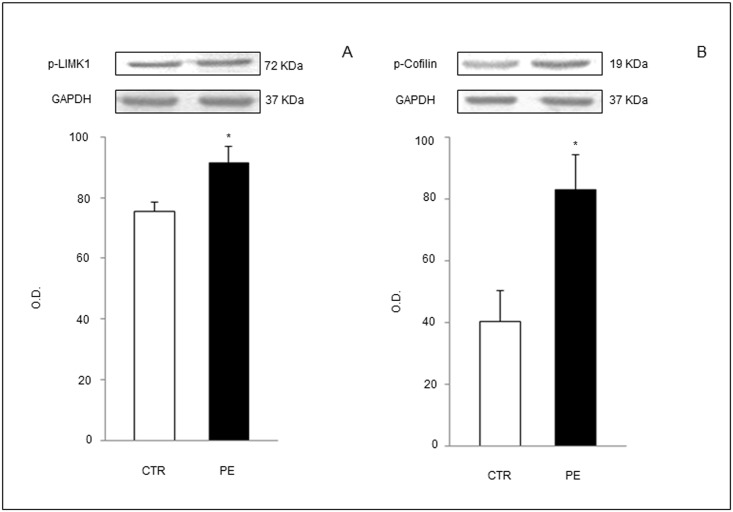
Western blot analysis of proteins involved in intracellular pathway initiated by D6 engagement by active ligands and leading to cofilin-inactivation and actin filaments depolymerization. Phosphorylated (p)-LIMK1 (A) and p-Cofilin (B) were increased in trophoblast cell lysates from PE patients compared to controls. GAPDH: glyceraldehyde-3-phosphate dehydrogenase (loading control). Results are expressed as mean ± SE of six experiments. *p<0.05.

WB analysis of trophoblast cell lysates from PE or CTR placentae showed increased phosphorylation of cofilin through LIMK1 phosphorilation cascade (Fig 3A and 3B), an intracellular signaling required for cofilin inactivation and actin network rearrangement, essential for D6 scavenging activity.

### CCL2 Chemokine Binding to Trophoblast Cells Is Reduced in PE

**Fig 4:**
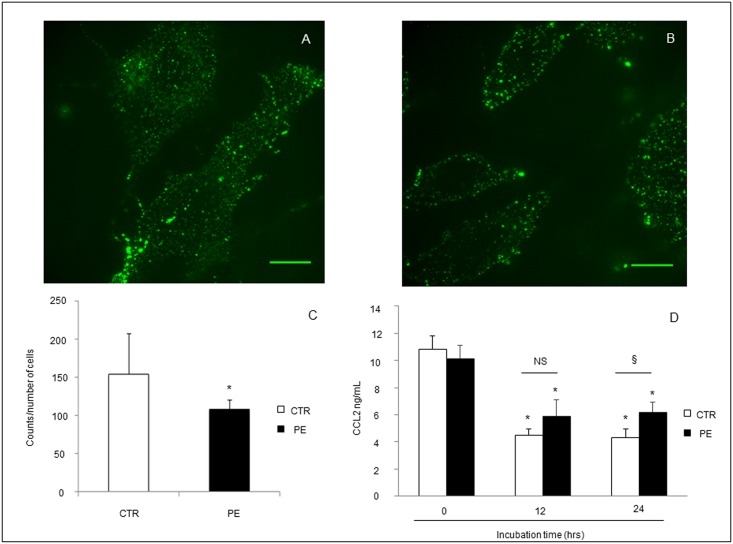
(A, B) Representative images of confocal analysis of CCL2 binding to D6 receptor in primary trophoblast cell cultures from normal pregnant women (A) or women with PE (B). Cells from controls (A) showed higher CCL2 binding to D6 (green) compared to PE (B) quantified by counting the number of fluorescent dots on the overall number of cells (C). Scale bar 20 μm. 4D. Functional assay of D6 receptor scavenging activity revealed, in both controls and PE cells, a significant scavenging of CCL2 after 12 and 24 hours of incubation (*p<0.05 *versus* time 0). Interestingly, atr 24 hours, decreased CCL2 scavenging activity was observed in cells from PE compared to CTR (§p<0.05 *versus* CTR). Results are expressed as mean ± SE of six experiments.

Confocal analysis of CCL2 binding to primary trophoblast cell culture from PE and controls revealed a lower intensity of signaling in PE compared to controls, suggesting a reduced D6 receptor availability to chemokines in PE (Fig 4A and 4B), although its increased mobilization on cell membranes, probably due to saturation by chemokines previously occurred on trophoblast cells *in vivo*.

### D6 Scavenging Activity Is Significantly Reduced in PE

Functional assay of D6 receptor scavenging activity in trophoblast cell cultures showed in both controls and PE cells a significant scavenging of CCL2 after 12 and 24 hours of incubation (*p<0.05 *versus* time 0) (Fig 4D). Interestingly, after 24 hours a significant decrease of CCL2 scavenging activity was observed in cells from PE compared to CTR (§p<0.05 *versus* CTR).

### Cytoskeleton Organization Is Impaired in PE Trophoblast Cells

**Fig 5:**
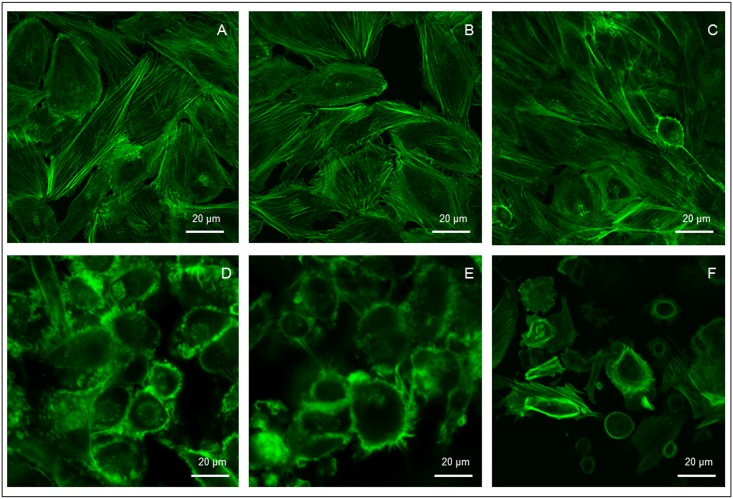
Confocal images of cytoskeleton F-actin fibers organization in primary trophoblast cell cultures from placentae of healthy pregnant women at term (A-C) or women with PE (D-F). In PE trophoblast cells display a dramatic disarrangement and loss of spatial orientation of actin fibers compared to controls. Scale bar 20 μm.

Confocal analysis of trophoblast cells cytoskeleton organization performed by intracellular F-actin FITC-staining showed a dramatic disarrangement and loss of spatial orientation of actin fibers in PE compared to controls (Fig 5).

## Discussion

D6 is a scavenger receptor that binds with high affinity CC chemokines, internalizes and targets the ligands for degradation [6,7]. It has been proposed to have a central role in controlling the inflammation at the maternal-fetal interface [22] since it is expressed in placenta on invading extravillous trophoblast and on the apical side of syncytiotrophoblast cells, at the very interface between maternal blood and fetus [21].

In fact, implantation process is associated with a robust maternal inflammatory response that is maintained throughout pregnancy and characterized by maternal leukocyte recruitment into the decidua [33,34]. This is thought to be a critical component of a successful pregnancy but, given the semi-allogenic nature of fetal tissues, mechanisms must also be in place to protect trophoblasts from maternal leukocyte attack.

This delicate balance may be compromised in pregnancy-related diseases such as PE, a placenta-induced disorder characterized by exacerbated systemic inflammatory response [3–5]. Indeed, higher circulating blood levels of pro-inflammatory cytokines, like CCL2, IL-6, IL-8, TNF-α and RANTES have been shown in PE [21, 23–25].

Particularly, Madigan and co-authors observed a trend of increase of circulating D6-binding chemokines levels in the third trimester of women later developing PE [21], although not reaching a significant difference, probably because of the small sample of patients and timing of blood sampling, not performed during overt PE.

Recently, Cho and coworkers showed decreased transcription and expression of D6 in placentae from 23 women with PE compared to 12 controls, suggesting a role of reduced placental expression of D6 in the exaggerated placental tissues inflammation typical of PE [35].

The aim of this study was to investigate circulating D6-binding chemokines levels in overt PE compared to term pregnancy controls and to compare, in the same women, placental expression and function of D6 decoy receptor.

Since we did not find significant differences in D6 placental expression between PE women and controls, neither in whole placental lysates nor in primary trophoblast cell lysates, we investigated possible differences in terms of D6 receptor engagement, activation and distribution in trophoblast cell cultures.

Herein, we reported, for the first time to our knowledge, that D6 decoy receptor is up-regulated in trophoblast cell membranes in PE compared to controls. This observation is consistent with our finding of increased circulating D6-chemokines in PE women compared to controls.

Indeed, in contrast to conventional chemokine receptors, after ligand engagement, D6 does not decrease but rather increases its expression on the cell surface, due to its mobilization from the intracellular pool, in particular by accelerating receptor recycling through the Rab11-positive vesicles [16]. Thus, constitutive cycling and ligand-dependent receptor up-regulation are mechanisms allowing rapid modulation of ligand uptake and degradation [36]. We are persuaded that higher circulating levels of pro-inflammatory chemokines in PE might massively bind to D6 decoy receptor at placental level and induce the scavenger up-regulation on trophoblast cell membranes, due to its mobilization from the intracellular pool.

Accordingly with increased trophoblast membrane expression of D6 receptor in PE, we observed a stronger activation of D6 intracellular LIMK1-cofilin signaling pathway, as a logical consequence of a higher chemokines-D6 binding on cell membranes, previously occurred in PE placentae *in vivo*.

In physiological conditions, phospholrylation, and thus, inactivation of cofilin would enable actin network rearrangements, critically required to mobilize the receptor from intracellular compartments, to increase in its abundance on the plasma membrane and its scavenging activity [18].

Interestingly, when adding exogenous CCL2 to primary trophoblast cell cultures, we noticed a reduced binding to cells obtained from PE placentae compared to those from normal pregnancies. This observation might be explained by the higher saturation of D6 receptor in PE trophoblast cells, probably due to local and systemic pro-inflammatory environment occurring in the syndrome. Surprisingly, when investigating D6 receptor from a functional point of view, we observed a reduced trophoblasts D6 scavenging ability in PE.

Since cytoskeleton is crucial for D6 intracellular trafficking and functionality, we studied F-actin organization in trophoblast cells and we discovered a dramatic disarrangement of cytoskeleton in PE compared to controls.Indeed, mobilization of D6 from intracellular endosomes and vesicles trafficking relies on the regulatory and motor proteins of the cytoskeleton [16,37]. Therefore, a dynamic actin cytoskeleton is required to sustain D6-mediated uptake and targeting of chemokine for degradation.

Overall, our results suggest that in all cases of PE, an impairment of trophoblast cells cytoskeleton, as a consequence of several potential injures, like syncytiotrophoblast oxidative stress [38], might occur and affect D6 function, that is, in the meantime, overstressed by increased D6-binding chemokines secretion. It is important to remark that syncytiotrophoblast stress, and thus cytoskeleton impairment, might take place, at different times, in both “placental” and “maternal” cases of PE, the first due to severe poor placentation, manifesting early in pregnancy and generally associated to intrauterine growth restriction, the last characterized by later onset and generally appropriate fetal growth, occurring when a placenta is working, supplying blood to the fetus, at the upper limits of its functional reserve [39].

The final effect can lead to impairment of cell cytoskeleton, that cannot allow neither chemokines degradation nor D6 receptor recycling, causing deficient regulation of inflammatory environment at maternal-fetal interface. This is consistent with the observation in trophoblast membranes isolated from PE placentae of higher expression of D6 but lower binding and scavenging of exogenously added CCL2. In fact, D6 mobilized to membrane surface for the increased levels of circulating chemokines, is mostly saturated by endogenous chemokines and is no more able, because of cytoskeleton injury, to degrade CCL2 or recycle intracellular D6 receptor.

These phenomena might result in increased inflammation-induced placental impairment and systemic inflammatory response, worsening placental function and, thus, maternal-fetal outcome in PE.

## Supporting Information

S1 Fig Western blot analysis of both D6 and the cytosolic marker GAPDH in trophoblast whole cells (A) or plasma membrane (B) lysates. The dramatic reduction of intensity of the GAPDH band in lysates of trophoblast plasma membrane samples (B) is an indirect proof of the effectiveness of membrane isolation procedure used in this study.
(TIF)

S2 Fig Confocal microscopy analysis D6 in trophoblast cells from a pre-eclamptic women. Sequenses of images acquired in the z—axis clearly show D6 distribution on trophoblast plasma membrane. A) D6 fluorescent staining; B) electronic transmission only; C) overlay. Scale bar 20 μm.
(TIF)
