# Supplementary figures and images for: Placental Chemokine Receptor D6 Is Functionally Impaired in Pre-Eclampsia
Source: PLoS One. 2016 Oct 25;11(10):e0164747. doi: 10.1371/journal.pone.0164747 (PMC5079655; doi:10.1371/journal.pone.0164747)

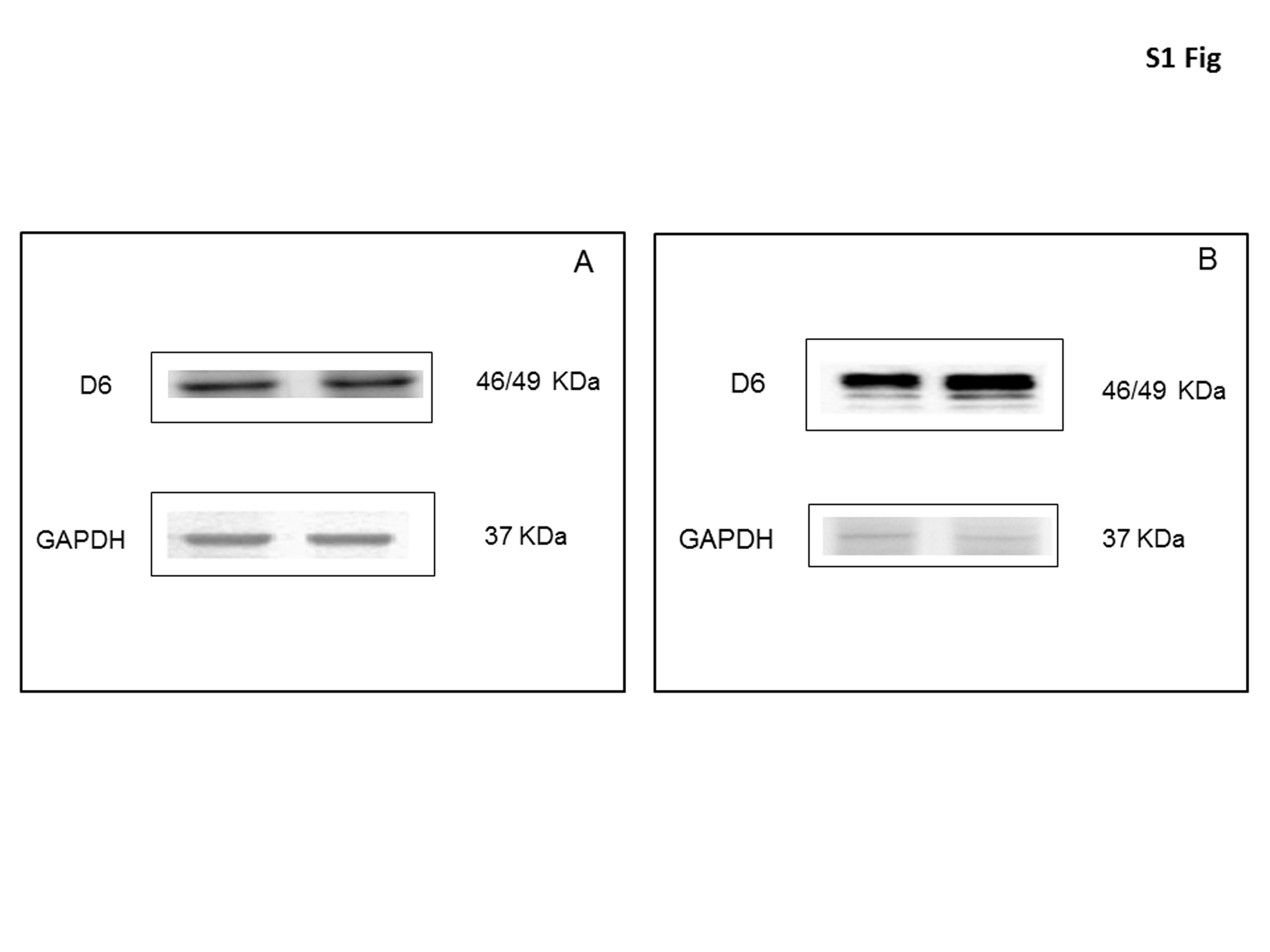

Supplement: S1 Fig — The dramatic reduction of intensity of the GAPDH band in lysates of trophoblast plasma membrane samples (B) is an indirect proof of the effectiveness of membrane isolation procedure used in this study. (TIF) [file pone.0164747.s001.tif]

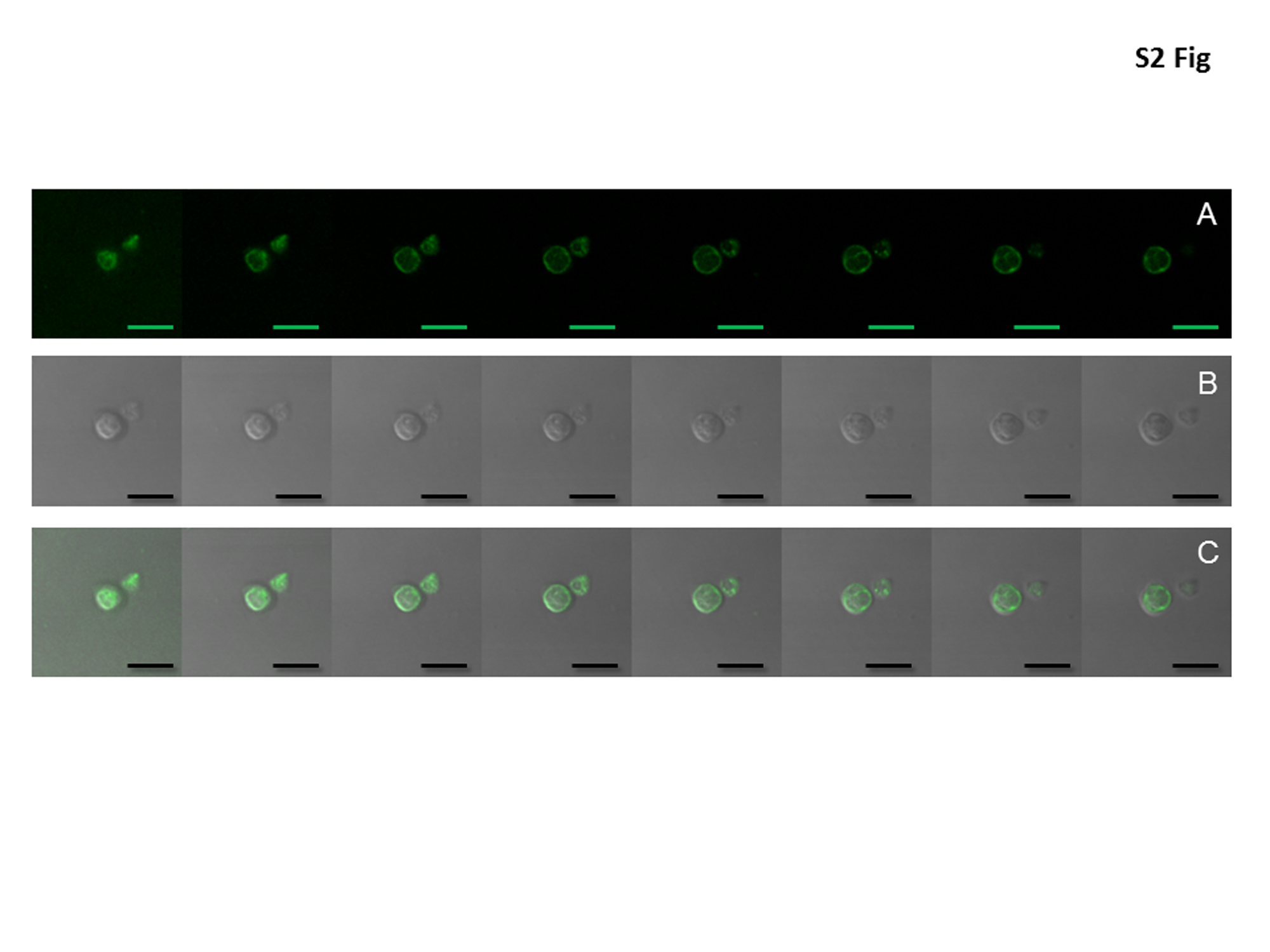

Supplement: S2 Fig — Sequenses of images acquired in the z—axis clearly show D6 distribution on trophoblast plasma membrane. A) D6 fluorescent staining; B) electronic transmission only; C) overlay. Scale bar 20 μm. (TIF) [file pone.0164747.s002.tif]
